# Applying Rprop Neural Network for the Prediction of the Mobile Station Location

**DOI:** 10.3390/s110404207

**Published:** 2011-04-08

**Authors:** Chien-Sheng Chen, Jium-Ming Lin

**Affiliations:** 1 Department of Information Management, Tainan University of Technology, No. 529, Jhongjheng Rd., YongKang Dist., Tainan City 71002, Taiwan; 2 Department of Communication Engineering, Chung-Hua University, No. 707, Sec.2, WuFu Rd., Hsinchu, Taiwan; E-Mail: jmlin@chu.edu.tw

**Keywords:** time of arrival (TOA), angle of arrival (AOA), back-propagation nseural network (BPNN), resilient back-propagation (Rprop)

## Abstract

Wireless location is the function used to determine the mobile station (MS) location in a wireless cellular communications system. When it is very hard for the surrounding base stations (BSs) to detect a MS or the measurements contain large errors in non-line-of-sight (NLOS) environments, then one need to integrate all available heterogeneous measurements to increase the location accuracy. In this paper we propose a novel algorithm that combines both time of arrival (TOA) and angle of arrival (AOA) measurements to estimate the MS in NLOS environments. The proposed algorithm utilizes the intersections of two circles and two lines, based on the most resilient back-propagation (Rprop) neural network learning technique, to give location estimation of the MS. The traditional Taylor series algorithm (TSA) and the hybrid lines of position algorithm (HLOP) have convergence problems, and even if the measurements are fairly accurate, the performance of these algorithms depends highly on the relative position of the MS and BSs. Different NLOS models were used to evaluate the proposed methods. Numerical results demonstrate that the proposed algorithms can not only preserve the convergence solution, but obtain precise location estimations, even in severe NLOS conditions, particularly when the geometric relationship of the BSs relative to the MS is poor.

## 1. Introduction

The problem of position determination of a mobile user in a wireless network has been studied extensively in recent years. It has received significant attention and various location identification technologies have been proposed in the past few years. A recent report and order issued by the U. S. Federal Communications Commission (FCC) in July 1996 requires that all wireless service providers provide the location information to emergency 911 (E-911) public safety services. The separate accuracy requirements of the E-911 mandate were set for network-based technologies: within 125 meters for 67 percent of calls, and within 300 meters for 95 percent of the calls. To date, satisfying the FCC accuracy requirement is very difficult. Most papers and their algorithms could not achieve this goal.

The various techniques proposed include signal strength (SS), angle of arrival (AOA), time of arrival (TOA) and time difference of arrival (TDOA). The signal strength scheme uses a known mathematical model to describe the path loss attenuation with distance. A fuzzy logic technique with a geometrical solution was applied to calculate range estimates through signal strength measurements [1]. The AOA scheme estimates the signal direction of arrival [2], to derive the mobile station (MS) location, by using either a directive antenna, or an antenna array leading to multiple lines of position. The TOA location scheme measures the propagation time for a radio wave to travel between the MS and a base station (BS). The TDOA scheme determines the position of MS by examining just the difference in time from MS to multiple BSs, rather than the absolute arrival time. Different potential applications of wireless location services have been well developed, including the E-911 subscriber safety services, location-based billing, fleet management and intelligent transportation system (ITS) [3].

One critical problem in wireless location systems is the non-line-of-sight (NLOS) propagation effect. A common requirement for high location accuracy is the presence of a line-of-sight (LOS) path between the MS and each participating BS. Due to the signal reflection or diffraction between MS and BSs, NLOS errors can significantly impact wireless location performance. Extensive research on NLOS effect mitigation for location estimation have been carried out in the past few years. Since in NLOS the delay has a higher variance than under LOS conditions, [4] proposed a decision framework to detect NLOS BSs’ via time series of estimates. An algorithm was proposed in [5] for TOA systems to mitigate NLOS effects by applying weights which are inversely proportional to their residuals for all possible BS combinations, then the NLOS BS with a larger residual has a lower effect on the MS location estimation. Similar residual schemes were proposed for both AOA systems in [6] and TDOA systems in [7]. The algorithms described in [5–7] perform well provided there are many available BSs being LOS with the MS in the system. Otherwise, they cannot improve the location accuracy. Based on the NLOS situation and how much *a priori* knowledge of the NLOS error is available, different NLOS identification and correction algorithms for mobile user location are proposed [8].

Another major concern that affects the choice of location scheme to deploy in cellular communication systems is *hearability* [9]. Here, *hearability* is the ability to receive signals from a sufficient number of BSs simultaneously at a sufficient power level [10]. It is difficult for the MS to receive signals from neighboring BSs in cellular communication systems, and the *hearability* is poor due to near-far effect and multiple access interference. [11] indicated the respective signal strength thresholds clearly show that the coverage in rural areas is much less than in urban areas. The likelihood of finding three BSs with received signal strength indication stronger than −100 dB is only 35 percent in rural areas, whereas it is about 84 percent in urban areas. It is difficult for an MS to detect three or more BSs for location purposes in rural areas. It is evident that the *hearability* in an IS-95 system is extremely poor [12]. The number of BSs that can be heard by a MS is only one if the MS is near its serving BS. Two or three BSs can be heard only at the edge of a cell. The insufficient number of available BSs limits the location-based services and impedes the implementation of location systems.

Due to poor *hearability*, it is reasonable to consider the hybrid methods by integrating two or more schemes. Comparing time-based methods with angle-based categories, both have their own advantages and limitations. In general, the AOA (angle-based) scheme requires only two BSs for location estimation, but it is necessary to deploy antenna array at BS for the AOA to work properly. In the case of time-based methods such as TOA and TDOA schemes, they require at least three BSs to be precisely located for a 2-D location estimation [13], nevertheless, they offer better accuracy than those of angle-based AOA schemes. The hybrid algorithm in [14] combining a TDOA technique with an additional AOA at the serving BS, can offer more accurate estimation in small error conditions. A hybrid TOA/AOA algorithm in [15], based on a nonlinear constrained optimization, can locate the MS even in NLOS environments for the case of three participating BSs. A hybrid range/range difference algorithm was used to estimate MS location in a global system for mobile communications (GSM) when only two BSs are available and the MS is located at the center of mass of the serving cell [16]. There is always an ambiguity in the MS location if only two TOA measurements are used. In order to resolve the ambiguity when an MS can be heard by only two BSs, both TOA and AOA information are required. Hence, we have proposed the hybrid TOA/AOA positioning methods for such *hearability*-constrained conditions in [17]. The MS is located at the various intersections of two circles and two lines, because the NLOS error always appears as a positive bias in the TOA measurements. Since the NLOS range errors are always positive, TOA measurements are greater than the true values. Therefore the true MS location should be inside the region enclosed by the overlap of the two circles. The above intersections are defined as feasible intersections. By using two AOA measurements to eliminate the least likely feasible intersection, the geometrical positioning methods are based on the weighted sum of the remaining feasible intersections enclosed by two TOA circles and two AOA line. These methods with different weighting algorithms can effectively eliminate the NLOS errors and provide more accurate positioning.

Artificial neural network (ANN) is an information processing method inspired by the biological nervous system, which can approximate nonlinear functions based on data sets. The system employs a set of activation functions and input-output of sample patterns that do not require *a priori* selection of a mathematical model. The back-propagation neural network (BPNN) is currently the most representative learning algorithm in ANN [18], and has been successfully applied to a wide range of scientific areas, especially in applications involving forecasting, image processing, pattern recognition and signal processing, and many others. BPNN continuously adjusts a set of weights of inputs and their corresponding outputs for the connections in the network to produce a mapping from input vectors to output vectors. It is an iterative algorithm using the gradient steepest descent method to minimize the error between the network actual output and desired output. This is particularly useful for those problems with an unknown optimal algorithm. The major drawbacks of the traditional BPNN are their slow learning process and have a tendency to be trapped into a local minimum.

Resilient back-propagation (Rprop) is the best algorithm in terms of convergence speed, accuracy as well as robustness with respect to the training parameters [19]. The Rprop is a local adaptive learning algorithm, the basic idea is to eliminate the harmful influence due to the weight step size of the partial derivative. Comparing to the back-propagation algorithm, the Rprop converges faster and needs less training. In this paper, we propose the algorithm based on Rprop to estimate MS location if both TOA and AOA measurements are simultaneously available from two BSs. We proposed two types for input data collection. The first type (divided type) establishes different input data subsets according to the number of the remaining feasible intersections. The second type (composite type) is to make the remaining feasible intersections in order. During the training period, the Rprop is trained to establish the nonlinear relationship between the remaining feasible intersections and the MS location. The remaining feasible intersections become the input data after training, and are passed through the trained Rprop to estimate the MS location. Simulation results show that the proposed algorithm always performs consistently better than Taylor series algorithm (TSA) [20,21], hybrid lines of position algorithm (HLOP) [22], and even the geometrical positioning methods proposed by us in [17]. Although it requires training, our algorithm are satisfied the FCC standard of accuracy in most of the NLOS error models. For both TSA and HLOP, the MS location accuracy can be critically affected by the relative geometry between BSs and MS. The proposed algorithm performs equally well for any MS location while TSA may not converge when the MS is located on the straight line passing through two of the BSs, and HLOP would produce a large location error when the measured angle is close to 90° or 270°.

The remainder of this paper is organized as follows. In Section 2, we describe the MS positioning methods by using TSA and HLOP. The geometrical positioning methods are reviewed in Section 3. Section 4 briefly describes BPNN and Rprop methods. In Section 5, we propose the algorithm based on Rprop to determine the position of the MS. Next, Section 6 compares the performance of the proposed algorithm with the other methods through simulation result. Finally, Section 7 draws conclusions.

## 2. Taylor Series Algorithm (TSA) and Hybrid Lines of Position Algorithm (HLOP)

**Figure 1.**
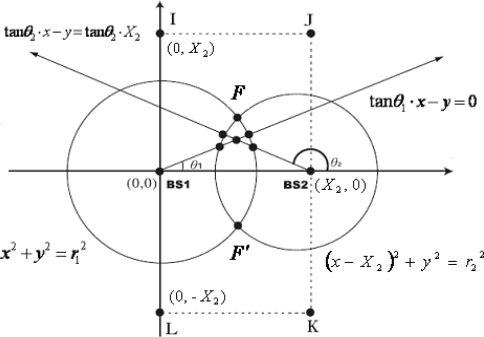
Geometric layout of the two circles and two lines.

If both the TOA and AOA measurements are accurate, then only one BS is required to locate the MS [17]. In reality, both TOA and AOA measurements contain errors due to NLOS propagation. Thus more than one BS is required for MS location of reasonable accuracy. Taking into account the constraint on *hearability*, the number of BSs available for estimating MS location is limited to two in this paper. However, each BS has both TOA and AOA measurement capabilities. Let *t_i_* denote the propagation time from the MS to BS*i*, *i* = 1, 2. The distances between BS*i* and MS can be expressed as:

(1) $$r_{i}=c\cdot t_{i}=\sqrt{{\left(x-X_{i}\right)}^{2}+{\left(y-Y_{i}\right)}^{2}}$$

where (*x*, *y*) and (*X_i_*, *Y_i_*) are the locations of MS and BS*i*, respectively. *c* is the propagation speed of the signals. If *θ_i_* is the angle between MS and BS*i*, with respect to a reference direction (x-axis), BS1 is located at (*X_1_*, *Y_1_*) = (0, 0), BS2 is located at (*X_2_*, *Y_2_*) = (*X_2_*, 0), and MS is located at (*x*, *y*), as shown in Figure 1, then *θ_i_* can be obtained as:

(2) $$\theta_{i}={\text{tan}}^{-1}\left(\frac{y-Y_{i}}{x-X_{i}}\right)$$

TSA [20,21] and HLOP [22] methods are commonly used to estimate the MS location, which are briefly described in this section.

### 2.1. Taylor Series Algorithm (TSA)

TOA and AOA measurements are used as inputs to the Taylor series position estimator. Let (*x*, *y*) is the MS location and (*x_v_*, *y_v_*) is the initially estimated position, let *x* = *x_v_* + *δ_v_* and *y* = *y_v_* + *δ_v_*. The MS location is obtained by linearizing the TOA and AOA equations through the use of a Taylor series expansion and retaining second-order terms, we have:

(3) Aδ≅z

where $A=\left[\begin{array}{cc} \kappa_{11} & \kappa_{12}\\ \kappa_{21} & \kappa_{22}\\ \phi_{11} & \phi_{12}\\ \phi_{21} & \phi_{22} \end{array}\right]$, $\delta =\left[\begin{array}{c} \delta_{x}\\ \delta_{y} \end{array}\right]$, $z=\left[\begin{array}{c} r_{1}-r_{v1}\\ r_{2}-r_{v2}\\ \theta_{1}-\theta_{v1}\\ \theta_{2}-\theta_{v2} \end{array}\right]$, and $\kappa_{i1}={\frac{\partial r_{i}}{\partial x}|}_{x_{v},y_{v}}$, $\kappa_{i2}={\frac{\partial r_{i}}{\partial y}|}_{x_{v},y_{v}}$, $r_{\mathrm{vi}}=\sqrt{{\left(x_{v}-X_{i}\right)}^{2}+{\left(y_{v}-Y_{i}\right)}^{2}}$, $\phi_{i1}={\frac{\partial \theta_{i}}{\partial x}|}_{x_{v},y_{v}}$, $\phi_{i2}={\frac{\partial \theta_{i}}{\partial y}|}_{x_{v},y_{v}}$, $\theta_{\mathrm{vi}}={\text{tan}}^{-1}\frac{y_{v}-Y_{i}}{x_{v}-X_{i}},\;i=1,\;2$.

The least-squares (LS) solution to the estimation problem is given by:

(4) $$\delta ={\left(A^{T}\;A\right)}^{-1}A^{T}\;z$$

It requires a proper initial position guess close to the true solution and can achieve high accuracy. This method is recursive and the computational overhead is intensive in the iteration. Due to the initial guess of the MS location is not accurate enough, the convergence of the iterative process is not assured [20,21]. In addition, to avoid the divergent problems in the simulations, the true MS location is used as an initial position.

### 2.2. Hybrid Lines of Position Algorithm (HLOP)

This scheme makes use of the original nonlinear range equations to produce linear lines of position (LOP), rather than circular LOP, to locate the MS. The method takes the advantage of simpler computation of MS location. The details of the linear LOP approach can be acquired by using the TOA measurements as in [23], and the hybrid linear LOP algorithm with AOA measurement in [22]. Combining the linear LOP and two AOA lines, the MS location can be determined by:

(5) Gl=h

where $l=\left[\begin{array}{c} x\\ y \end{array}\right]$ denotes the MS location, $G=\left[\begin{array}{cc} X_{2} & 0\\ \text{tan}\;\theta_{1} & -1\\ \text{tan}\;\theta_{2} & -1 \end{array}\right]$ and $h=\frac{1}{2}\left[\begin{array}{c} r_{1}^{2}-r_{2}^{2}+X_{2}{}^{2}\\ 0\\ 2\left(X_{2}\cdot \text{tan}\;\theta_{2}\right) \end{array}\right]$.

Again, the LS solution to Equation (5) is given by:

(6) $$l={\left(G^{T}\;G\right)}^{-1}G^{T}\;h$$

## 3. Geometrical Positioning Methods

From the viewpoint of geometric approach, the TOA value measured at any BS can be used to form a circle centered at the BS. The MS position is then given by the intersection of the circles from multiple TOA measurements. Similarly, a single AOA measurement constrains the MS along a line. Each of the following equations describes a circle for TOA, a line for AOA, as shown in Figure 1:

(7) $$\text{Circle 1}:\;x^{2}+y^{2}=r_{1}{}^{2}$$

(8) $$\text{Circle 2}:\;{\left(x-X_{2}\right)}^{2}+y^{2}=r_{2}{}^{2}$$

(9) $$\text{Line 1}:\;\text{tan}\;\theta_{1}\cdot x-y=0$$

(10) $$\text{Line 2}:\;\text{tan}\;\theta_{2}\cdot x-y=\text{tan}\;\theta_{2}\cdot X_{2}$$

If there is no error or even no noise at all, the circles and lines will intersect at only one point. However, this is usually not the case in practice where the NLOS effect exists. NLOS propagation is quite common and it seriously degrades location accuracy. The intersections of two TOA circles and two AOA lines will be spread over a region, which will be offset from the true MS location. Because of the fact that NLOS effect always increases the propagation delay, the measured TOA estimated are always greater than the true values due to the excess path length. The true MS location must lie in the region of overlap of the two circles. As mentioned earlier, the intersecting points that are within this are defined as feasible intersections. Hence, the feasible intersections must satisfy the following inequalities simultaneously:

(11) $$x^{2}+y^{2}\leq r_{1}{}^{2}$$

(12) $${\left(x-X_{2}\right)}^{2}+y^{2}\leq r_{2}{}^{2}$$

The most direct method is to utilize these feasible intersections of the circles and lines to estimate the MS location. To achieve high accuracy of MS location with less complexity, we have proposed a class of geometrical positioning methods in [17] and outlined as follows.

### 3.1. Averaging Method

By using two AOA measurements, the least likely intersection is first eliminated. The MS location is obtained to calculate the average value of all the remaining AOA measurements with feasible intersections.

Step 1. Find all the feasible intersections of the two circles and two lines.
Step 2. Assume *F* and *F′* are the intersections of the two circles as shown in Figure 1, *F′* is considered to be the least likely intersection if 0° < *θ*_1_, *θ*_2_ < 180°, and *F* is considered to be the least likely intersection if 180° < *θ*_1_, *θ*_2_ < 360°. Delete the least likely intersection from the set of feasible intersections and there will be *N* remaining feasible intersections.
Step 3. The MS location (*x̄_N_*, *ȳ_N_*) is estimated by averaging these remaining feasible intersections, where:
  (13) $${\bar{x}}_{N}=\frac{1}{N}\sum_{i=1}^{N}x_{i}\text{ and }{\bar{y}}_{N}=\frac{1}{N}\sum_{i=1}^{N}y_{i}$$

### 3.2. Distance-Weighted Method

However, not all the remaining feasible intersections can always provide information of the same value for location estimation. In this method, the weights are inversely proportional to the squared value of the distance between the remaining feasible intersections and the average MS location.

Steps 1–3 are the same as those of the averaging method.
Step 4. Calculate the distance *d_i_* between each remaining feasible intersection (*x_i_*, *y_i_*) and the average location (*x̄_N_*, *ȳ_N_*):
  (14) $$d_{i}=\sqrt{{\left(x_{i}-{\bar{x}}_{N}\right)}^{2}+{\left(y_{i}-{\bar{y}}_{N}\right)}^{2}},\;1\leq \;i\leq N$$
Step 5. Set the weight for the *i*th remaining feasible intersection to ${\left(d_{i}^{2}\right)}^{-1}$. Then the MS location (*x_d_*, *y_d_*) is determined by:
  (15) $$x_{d}=\frac{\sum_{i=1}^{N}{\left(d_{i}{}^{2}\right)}^{-1}\cdot x_{i}}{\sum_{i=1}^{N}{\left(d_{i}{}^{2}\right)}^{-1}}\text{ and }y_{d}=\frac{\sum_{i=1}^{N}{\left(d_{i}{}^{2}\right)}^{-1}\cdot y_{i}}{\sum_{i=1}^{N}{\left(d_{i}{}^{2}\right)}^{-1}}$$

One can see in the averaging method and distance-weighted method, all the remaining feasible intersections will affect the MS location estimation. In the following we also propose two methods of sort averaging and sort-weighted, which can be applied without considering the influence of feasible intersections for too far away from the average MS location.

### 3.3. Sort Averaging Method

Steps 1–4 are the same as those of the distance-weighted method.
Step 5. Rank the distances *d_i_* in increasing order and re-label the remaining feasible intersections in this order.
Step 6. The MS location (*x̄_M_*, *ȳ_M_*) is estimated by the mean of the first *M* remaining feasible intersections:
  (16) $${\bar{x}}_{M}=\frac{1}{M}\sum_{i=1}^{M}x_{i},\;{\bar{y}}_{M}=\frac{1}{M}\sum_{i=1}^{M}y_{i}\;\;\left(M=0.5*N\leq N\right)$$

### 3.4. Sort-Weighted Method

Steps 1–5 are the same as those of the sort averaging method.
Step 6. The MS location is estimated by a weighted average of the first *M* remaining feasible intersections with weight = ${\left(d_{i}^{2}\right)}^{-1}$:
  (17) $$x=\frac{\sum_{i=1}^{M}{\left(d_{i}{}^{2}\right)}^{-1}\cdot x_{i}}{\sum_{i=1}^{M}{\left(d_{i}{}^{2}\right)}^{-1}},\;y=\frac{\sum_{i=1}^{M}{\left(d_{i}{}^{2}\right)}^{-1}\cdot y_{i}}{\sum_{i=1}^{M}{\left(d_{i}{}^{2}\right)}^{-1}}\;\;\left(M=0.5*N\leq N\right)$$

### 3.5. Threshold Method

The weight of this method is based on how close the remaining feasible intersections are. Those feasible intersections that are closer to one another are assigned with greater weights. In other words, those intersections that are in close proximity will be assigned with greater weights.

Steps 1 and 2 are the same as those of the averaging method.
Step 3. Calculate the distance *d_mn_*, 1 ≤ *m*, *n* ≤ *N*, between any pair of feasible intersections.
Step 4. Select a threshold value *D_thr_* as the average of all the distances *d_mn_*.
Step 5. Set the initial weight *I_k_*, 1 ≤ *k* ≤ *N*, to be zero for all remaining feasible intersections.
  If *d*_*mn*_ ≤ *D*_*thr*_, then *I*_*m*_ = *I*_*m*_ + 1 and *I*_*n*_ = *I*_*n*_ + 1 for 1 ≤ *m, n* ≤ *N*.
Step 6. The MS location (*x_t_*, *y_t_*) is estimated by:
  (18) $$x_{t}=\frac{\sum_{i=1}^{N}I_{i}\cdot x_{i}}{\sum_{i=1}^{N}I_{i}}\;\;\text{and }\;y_{t}=\frac{\sum_{i=1}^{N}I_{i}\cdot y_{i}}{\sum_{i=1}^{N}I_{i}}$$

## 4. The Traditional BPNN Algorithm and the Rprop Algorithm

### 4.1. The Traditional BPNN Algorithm

In this section, we describe the methodology based on artificial neural network (ANN). It is a technique that models the learning procedures of a human brain, and employs a set of activation functions, either nonlinear or linear, thus one doesn’t require *a priori* selection of a mathematical model. Further, this method has been proved to be very useful for various applications. One of the most influential developments in ANN was the invention of the BPNN, which provides advantages of non-linear problem solving ability. BPNN is a multi-layered, feed-forward architecture with supervised learning method for computer learning and modeling. A supervised feed-forward neural network can not only learn from the training data to discover patterns representing the input and output variables, but approximate many problems with high accuracy. In a supervised learning approach, a set of input variables is used for which the corresponding output variables are known.

**Figure 2.**
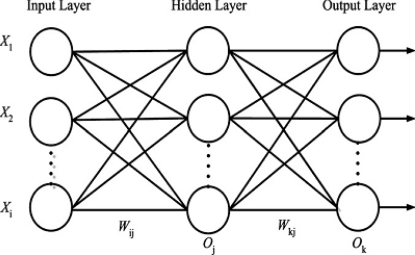
A fully connected multilayer feed-forward network with one hidden layer.

Generally speaking, the BPNN architecture comprises one input layer, one output layer, with one or a number of hidden layers in between them. Although a network with multiple hidden layers is possible, a single layer is sufficient to model arbitrarily complex nonlinear functions. With proper selection of architecture, it is capable of approximating most problems with high accuracy and generalization ability. The input layer receives information from the external sources and passes this information to the network for processing. The hidden layer determines the mapping relationships between neurons are stored as weights of connecting links. When the input and output variables are related nonlinearly, the hidden layer can extract higher level features and facilitate generalization. The output from the output layer is the prediction of the net for the corresponding input. The structure of BPNN chosen for the present problem is shown in Figure 2. Each layer consists of several neurons and the layers are interconnected by sets of correlation weights. A standard ANN comprises numerous simple processing units called neurons. Each node is connected to other neurons through directed connecting links; each neuron is a processing unit that contains an activation function and an associated weight. The active function is mathematical formula and used to transform the output such that it falls within an acceptable range. In this paper, the activation functions of hidden layer and output layer are hyperbolic tangent sigmoid function and linear transfer function. A weight returns a mathematical value for the relative strength of connections to transfer data from one layer to another layer.

BPNN estimate relation between input and output of sample patterns by updating iteratively the weights in the network so as to minimize the difference between the actual output vectors and the desired output vectors. The back propagation learning algorithm is composed of initialization, a forward pass, and a backward pass. The weights and biases in the network are initialized to small random numbers. Once these parameters have been initialized, the network is ready for training. A training pattern consists of a set of the input vectors and the corresponding output vectors. In the beginning, a set of training patterns are fed to the input layer of the network. The forward pass starts from the input layer, the net inputs of the neurons are multiplied with corresponding weights, then summated, and transferred to the hidden layer. The activated signals are outputted from the hidden layer, and are passed forward to the output layer. Finally, the output of BPNN is generated. Subsequently in the backward pass, the error between actual output and desired output is calculated. The error function Ψ is defined as the mean squared sum of differences between the actual output vector *T_k_* and the desired output vector *O_k_*:

(19) $$\Psi =\frac{1}{2}\sum_{k}{\left(T_{k}-O_{k}\right)}^{2}$$

The error signal at the output layer is propagated backward to the input layer through the hidden layer in the network. Back-propagation is so named because the error derivatives are calculated in the opposite direction of signal propagation. In the training process, the gradient descent method calculates and adjusts the weight of the network to minimize the error. In the weight updating algorithm, the derivative of the error with respect to the weight was first negated then multiplied by a small constant β known as the learning rate, as expressed in the following equation:

(20) $$\Delta w_{\mathrm{ij}}{}^{\left(t\right)}=-\beta \frac{\partial \Psi^{\left(t\right)}}{\partial w_{\mathrm{ij}}}$$

The negative sign indicates that the new weighting vector is moving in a direction opposite to that of the gradient. In the learning process of neural network, the learning rate affects the speed of convergence. The training process may lead to an oscillatory state if a learning rate is too fast, on the other hand, the convergence speed may suffer if the learning rate is too slow. The training process may not converge in the case of either a too high or too low value for the learning rate *β*. To accelerate the convergence, a momentum *α* can be added to the learning procedures [24]:

(21) $$\Delta w_{ij}{}^{\left(t\right)}=-\beta \frac{\partial \Psi^{\left(t\right)}}{\partial w_{\mathrm{ij}}}+\alpha \Delta w_{\mathrm{ij}}^{\left(t-1\right)}$$

**Figure 3.**
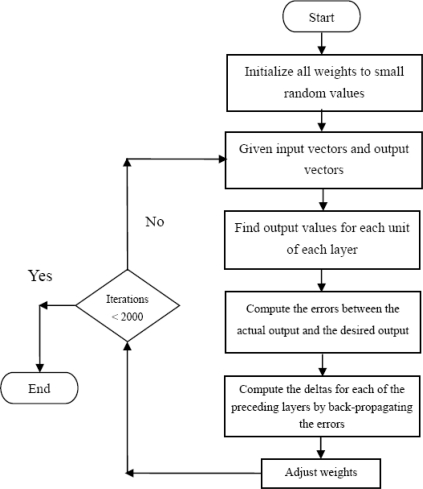
The flow chart of the calculation procedure for BPNN.

In Equation (21) *α* is between 0 and 1. When *α* = 0, a weight change is completely dependent on the value of gradient. When *α* = 1, the amount of new weight change is set to that of the last weight change and the gradient is simply ignored. The weights are adjusted to make the actual output move closer to the desired output and to obtain the final outputs. This process is repeated until the error is less than a pre-specified level for each of the training data points, or a large number of training iterations have already been run. In summary, the flow chart of training procedure of BPNN is in Figure 3 and the steps are listed as follows.

Set the number of the layer and the number of neurons in each layer:

1. Set *β*, *α* and initial values of the weights, and the biases in the network are initialized to small random numbers.
2. Giving input and output vectors.
3. Compute the output values of each layer and unit in a feed-forward direction.
  - Calculate the output for the *j*th hidden neuron.
  - Calculate the output for the *k*th output neuron.
4. Calculate the error function at the output neuron.
5. Compute the deltas for each of the preceding layers by back propagating the errors.
  - Calculate error for the *k*th output neuron.
  - Calculate error for the *j*th hidden neuron.
6. Update all weights and biases
7. Repeat steps 3–7 until the iteration has finished or the algorithm is convergent.

### 4.2. Rprop Algorithm

Compared to the traditional BPNN algorithm, the Rprop algorithm can provide faster training and rate of convergence, and has the capability to escape from local minima. The Rprop is known to be very robust with respect to their internal parameters and therefore regarded as one of the best first-order learning methods among the ANN algorithms. Rprop is a first-order algorithm and its time and memory requirement scale is linear with the number of parameters to optimize. The Rprop algorithm is probably the most easily adjustable learning rule, slight variations of the values of parameters can not affect the convergence time. The activation function of the hidden and output layers is treated as linear transfer function. Rprop is easy to implement and the hardware implementation is described in [25]. Comparing to back-propagation, one of the advantages of Rprop algorithm is that the magnitude of the partial derivative does not affect weight update and it depends only on the signs of the partial derivative. Thus it allows for faster convergence than the back-propagation can do. Rprop performs a direct adaptation of the weighting step based on local gradient information. A crucial difference to the previously developed adaptation techniques is that the adaptation effort won’t be blurred by the gradient behavior. The main idea of Rprop is to reduce the potential spurious effect of the partial derivative on weight-updates by retaining only the sign of the derivative as an indication of the direction in which the error function will be changed by the weight-update. We introduce an individual update-value Δ*_ij_*(*t*) for each weight, which solely determines the size of the weight-update. This adaptive update-value evolves during the learning process based on its local sight on the error function Ψ, according to the following learning rule [19]:

(22) $$\Delta_{ij}^{\left(t\right)}=\{\begin{array}{l} \eta^{+}\cdot \Delta_{\mathrm{ij}}^{\left(t-1\right)}\;,\text{ if }{\frac{\partial \Psi }{\partial w_{\mathrm{ij}}}}^{\left(t-1\right)}\cdot \frac{\partial \Psi^{\left(t\right)}}{\partial w_{\mathrm{ij}}}>0\\ \eta^{-}\cdot \Delta_{\mathrm{ij}}^{\left(t-1\right)}\;,\text{ if }{\frac{\partial \Psi }{\partial w_{\mathrm{ij}}}}^{\left(t-1\right)}\cdot \frac{\partial \Psi^{\left(t\right)}}{\partial w_{ij}}<0\\ \;\Delta_{\mathrm{ij}}^{\left(t-1\right)}\;,\;\mathrm{else} \end{array}$$

where 0 < *η*^−^ < 1 < *η*^+^. We can simply describe the adaptation rule as follows: Whenever the partial derivative of the error function ψ with respect to the corresponding weight *w_ij_* changes its sign, it indicates that the value of last update was too big and the algorithm has jumped over a local minimum. The update-value Δ*_ij_* is decreased by a factor *η*^−^. If the derivative retains its sign, the update-value is slightly increased by the factor *η*^+^ in order to accelerate convergence in shallow regions.

Once the update-value for each weight is adapted, the weight-update itself follows a very simple rule: if the derivative is positive (increasing error), the weight is decreased by its update-value, if the derivative is negative, the update-value is added to the weight:

(23) $$\Delta w_{\mathrm{ij}}^{\left(t\right)}=\{\begin{array}{l} -\Delta_{\mathrm{ij}}^{\left(t\right)}\;,\text{ if }{\frac{\partial \Psi }{\partial w_{\mathrm{ij}}}}^{\left(t\right)}>0\\ +\Delta_{\mathrm{ij}}^{\left(t\right)}\;,\text{ if }{\frac{\partial \Psi }{\partial w_{\mathrm{ij}}}}^{\left(t\right)}<0\\ \;0\;,\;\mathrm{else} \end{array}$$

There is one exception to the rule above. If the partial derivative changes sign, *i.e*., the previous step was too large and the minimum was missed, the previous weight-update is reverted:

(24) $$\Delta w_{\mathrm{ij}}{}^{\left(t\right)}=-\Delta w_{\mathrm{ij}}^{\left(t-1\right)},\text{ if }\frac{\partial \Psi^{\left(t\right)}}{\partial w_{\mathrm{ij}}}\cdot \frac{\partial \Psi^{\left(t-1\right)}}{\partial w_{\mathrm{ij}}}<0$$

Due to that ‘backtracking’ weight step, the derivative is supposed to change its sign once again in the following step. In order to avoid a double punishment of the update value, there should be no adaptation of the update value in the succeeding step. In practice this can be done by setting ∂ψ^(*t*−1)^/∂*w*_*ij*_ = 0 in the Δ*_ij_* update-rule above.

## 5. Proposed Location Algorithm Based on Rprop

To improve the accuracy of MS location, we proposed the employment of Rprop, a supervised learning neural network to obtain an approximation of MS location. The remaining feasible intersections are fed to the input layer, and MS location is the only one variable in the output layer. Given a number of known input-output training patterns, the Rprop models are trained continuously and deployed to adjust the weights with one hidden layer. A trained Rprop is to minimize the difference between the actual MS location and the desired MS location. The network has the following input-output mapping:

Input: V remaining feasible intersections (V = 1, 2,…, 6)
Output: desired MS location

**Figure 4.**
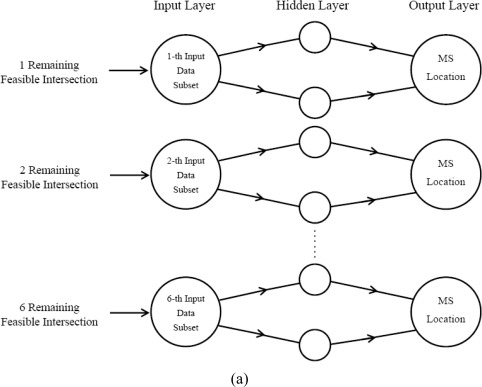

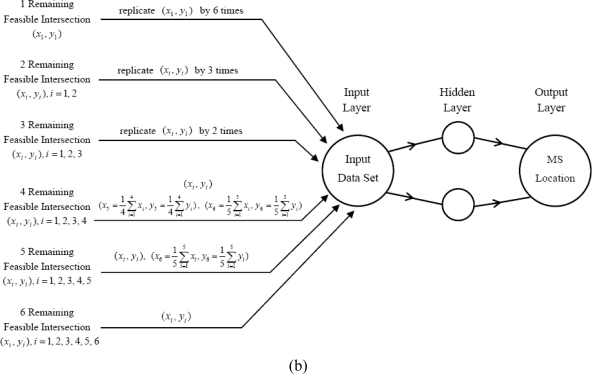
Structure of the prediction models for **(a)** Type 1 (Divided Type) and **(b)** Type 2 (Composite Type).

The number of the remaining feasible intersections depends on the geometric relationship of the two TOA circles and two AOA lines. In this case, the number of the remaining feasible intersections is between 1 and 6. Every measurement will result in one input data. Figure 4 illustrates the structure used in Rprop MS location forecasting model of a three-layered network. Two types of input layer for training purpose are identified and explained in detail as follows:

### Type 1 (Divided Type):

According to the number of the remaining feasible intersections, the first type establishes different input data subsets respectively. For each measurement, we collect the V remaining feasible intersections and put them into the V-th input data subsets separately. There are six data subsets in this input layer for training purpose, and the measurement number of each subset won’t be identical. From simulation results, the measurement number of 4 remaining feasible intersections is the maximum, while the measurement number of 1 remaining feasible intersections is the minimum.

The detailed steps are as follows:

1. Collect the V remaining feasible intersections of two TOA circles and two AOA lines. (V = 1, 2,…, 6).
2. If the number of remaining feasible intersection is V, then placing these V points in the V-th subset. The V remaining feasible intersections are belonging to the corresponding V-th input data subsets separately.
3. The 6 input data subsets with various measurement numbers are trained according to Rprop.

### Type 2 (Composite Type):

The second type is a collection of the V remaining feasible intersections in order. Regardless of the number of remaining feasible intersections in each measurement, we will only establish one input data set. The summation of all the measurement number for the 6 subsets is equal to the number of all measurements. The detailed steps are as follows:

1. Collect the V remaining feasible intersections for each measurement and expand to six ones in a data set.
2. The method to expand the remaining feasible intersections to 6 ones during each measurement is as follows.
  - If the number of remaining feasible intersections is V, replicate them by (6/V) times. (V = 1, 2, 3)
  - If the number of remaining feasible intersections is 4, take the average value of these 4 points and treat it as the fifth point. By this manner the 6th point is the average of the 5 previous numbers.
  - If 5 remaining feasible intersections are collected, take an average of these 5 points as the 6th one.
3. After expansion, placing the 6 remaining feasible intersections in the input data set for training purposes.

The training data is different from the data that uses to estimate the MS location. That is, the training input-output patterns is no longer be used after training is down. In real application, we collect the remaining feasible intersections and the desired MS location to train the neural network prior to the practical use. After the training, then the remaining feasible intersections as input data (with the MS locations be unknown) can not only pass through the trained Rprop more quickly, but estimate the better appropriate MS location. Whenever we start to find the positions, the “remaining feasible intersections” can be used as the trained input, as we expect this model can estimate MS locations quickly and precisely. In addition, we have found that when there are only 200 pieces of input-output patterns as training data, the proposed algorithm still work better than the other methods. Therefore, the conclusion is that the proposed algorithm can be applied in practical situations.

## 6. Simulations Results

In this section for fairly comparison with various methods we apply the computer simulations to demonstrate the performance of the proposed algorithm. In the simulations, the BSs are respectively located at (0, 0) and (2,000 m, 0). Each simulation is performed by 10,000 independent runs, and the MS location is chosen randomly according to a uniform distribution within the rectangular area formed by the points I, J, K and L, as shown in Figure 1. To validate the effectiveness of the proposed algorithm, the remaining feasible intersections and the desired MS location are collected for comparison. The proposed hybrid TOA/AOA algorithm employs Rprop for MS location estimation and performance evaluation. Regarding the NLOS effects, three propagation models are adopted, namely, the uniformly distributed noise model [15], the biased uniform random model [22], as well as the distance-dependent model [15].

The first NLOS propagation model is based on the uniformly distributed noise model [15], in which the TOA measurement error is assumed to be uniformly distributed over (0, *U_i_*), for *i* = 1, 2 where *U_i_* is the upper bound and the AOA measurement errors are assumed to be *f_i_* = *w*·5° and *f_i_* = *w*·10°, where *w* is a uniformly distributed variable over [−1, 1] [26]. Before applying Rprop to estimate MS location, the parameter must be set in advance, such as the numbers of hidden neurons, and training iterations (epochs). The parameter settings for network architectures must be determined carefully to avoid constructing a worse network model; otherwise, they may increase the computational cost and produce worse results. Trial-and-error methods are used to determine the parameter settings for network architectures. We attempted to find the optimal parameter as well as maintain good performance at the same time.

**Figure 5.**
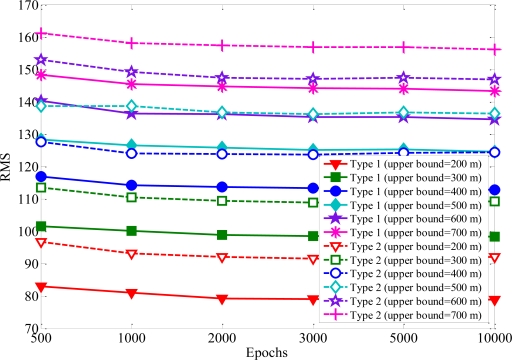
RMS errors reduction according to the number of epochs.

**Figure 6.**
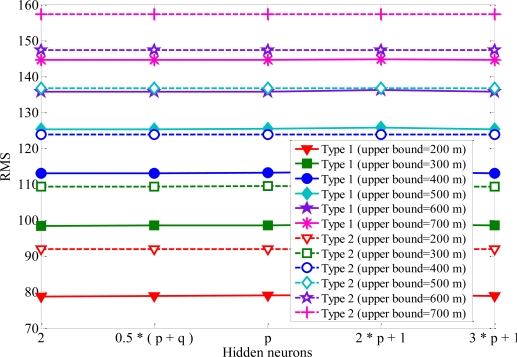
The RMS errors with various neurons numbers of hidden layer.

Single hidden layer is the most widely used one among various learning methods for neural networks. It is well enough to model arbitrarily complex nonlinear functions [27]. Therefore, the number of hidden layers is set at one. To examine how close the forecast to the real MS location, the root-mean-square (RMS) error is employed to evaluate the performance of the proposed algorithm. If the network outputs are relatively close to the real MS location, RMS error will have small values. Figure 5 shows the RMS error of convergence *versus* the increased number of epochs. One can see the Rprop with one hidden layer can map the remaining feasible intersections to MS location. At the beginning of the training period, the error is reduced rapidly. When the number of epochs is above 2,000, it will not give further performance improvement. Some general rules to determine the number of hidden neurons are: (i) 0.5 (*p* + *q*), (ii) *p*, (iii) 2*p* + 1, (iv) 3*p* + 1, where *p* and *q* are input neurons and output neurons, respectively [28]. The RMS errors with various numbers of hidden-layer neurons are listed in Figure 6 for comparison. One can see the RMS error converged to the same minimum value for various hidden layer neurons. The accuracy of MS location is hardly affected by the numbers of hidden-layer neurons. The number of neurons in the hidden layer is set to be 2 because of the satisfactory prediction performance. In order to avoid increasing the computation load, 2 hidden neurons and 2,000 training iterations are used in the simulations.

**Figure 7.**
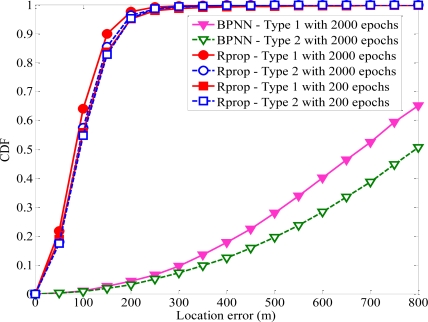
Comparison of average MS location based on BPNN and Rprop.

If BS1 is the serving BS of MS, its TOA and AOA measurements should be more accurate. The variables of this model are chosen as follows: *U*_1_ = 200 m, *U*_2_ = 400 m, *τ*_1_ = 5° and *τ*_2_ = 10°. The proposed algorithm based on Rprop algorithm produce more accurate estimations of MS location than those based on BPNN with a learning rate of 0.01, as shown in Figure 7. We can see that BPNN-based with 2,000 epochs has the worst performance and Rprop-based with 2,000 epochs has the best performance. Even Rprop-based with 200 epochs perform better than BPNN-based with 2,000 epochs. The number of epochs of Rprop is less than the traditional BPNN. So Rprop method can offer much faster convergence and require much less convergence time. The results show that Rprop algorithm can accurately estimate MS location. The superior performance for the Rprop algorithm has been demonstrated by comparing the RMS errors. One can see that Rprop algorithm produces more accurate estimations of MS location than traditional BPNN algorithm. Therefore, we select Rprop algorithm to estimate MS location in this paper.

**Figure 8.**
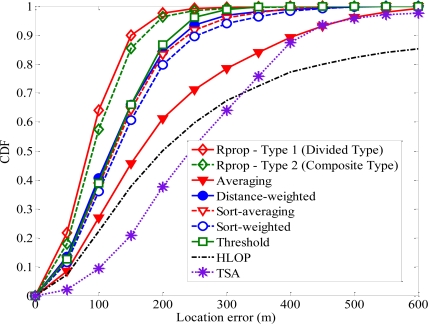
Comparison of error CDFs when NLOS errors are modeled as the upper bound.

Figure 8 shows cumulative distribution functions (CDFs) of the location error for different methods based on uniformly distributed noise model. To check for convergence, the initial guess of MS location in TSA is chosen to be the true solution in our simulations. Simulations demonstrated that at least three iterations are required for TSA to converge. We can see that TSA and HLOP offer the worst performance, and the proposed algorithm has good ability to make more accurate estimations of MS location. The divided type with six input data subsets for training performs better than the composite type. Thus the proposed algorithm can always yield better performance, compared with TSA, HLOP and the other geometrical positioning methods.

**Figure 9.**
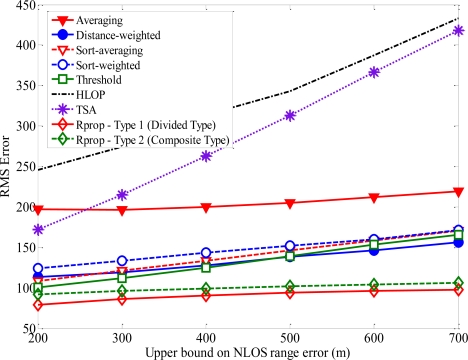
Performance comparison of the location estimation methods when the upper bound is used to model the NLOS.

Based on the proposed neural network structure stated above, the Rprop can be applied to estimate the location of MS for every input data. Figure 9 shows how the average location error is affected by the upper bound on NLOS error. The upper bound of NLOS error for BS1 is 200 m and those of other BSs range from 200 m to 700 m. After the training period, the superior performance for the proposed algorithm can be proved by comparing the RMS error of MS location. In general, as the upper bound on NLOS error increases, then the average magnitudes of the NLOS errors will also increase, which leads to less accurate location estimation. Note that the proposed algorithm can deal with large errors more effectively than the other methods. For most of the upper bound on NLOS errors, the average location errors for TSA and HLOP are larger than at least twice that for the proposed algorithm. The sensitivity of the proposed algorithm with respect to the NLOS effect is much less than those of TSA and HLOP methods.

**Figure 10.**
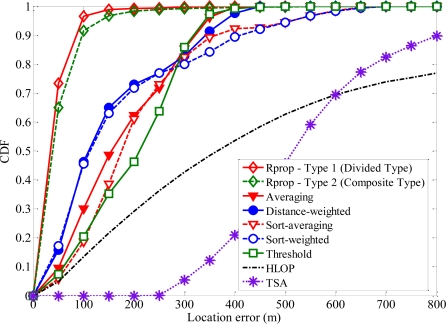
CDFs of the location error with distance-dependent NLOS error.

The second NLOS propagation model is based on the distance-dependent NLOS error model [15]. The NLOS range error for the *i*th range is taken to be *ξ_i_ = χ_i_*·*R_i_*, where *χ_i_* is a proportional constant and *R_i_* is the true range between *i*-th BS and MS [15]. It makes intuitive sense to view NLOS errors as being proportional to the distance traveled by the signal. The AOA measurement error is assumed to be *f_i_* = *w*·*τ_i_*, for *i* = 1, 2 [26]. The variables are chosen as follows: *χ*_1_ = 0.13, *χ*_2_ = 0.2, *τ*_1_ = 2.5° and *τ*_2_ = 5° Figure 10 shows the CDF of the average location error of different algorithms with distance-dependent NLOS error. Compared with the other methods, the accuracy of MS location was considerably improved with the proposed algorithm. We can see that TSA has the worst performance, and the proposed algorithm with divided type has the best performance, followed by the proposed algorithm with composite type.

**Figure 11.**
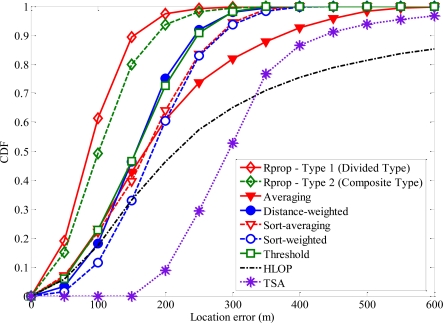
Comparison of location error CDFs with biased uniform random error.

The third NLOS propagation model is based on a biased uniform random variable [22], in which the measured error of TOA between MS and BS*i* is assumed to be *γ_i_* = *ρ_i_* + *w*·*μ_i_*, for *i* = 1, 2 where *ρ_i_* and *μ_i_* are constants. Similarly, the measured error of AOA, is modeled as |*a_i_*| = *b_i_* + *w*·*c_i_*, for *i* = 1, 2 where *b_i_* and *c_i_* are constants. The error variables for the two BSs are chosen as follows: *ρ*_1_ = 50 m, *ρ*_2_ = 150 m, *μ*_1_ = *μ*_2_ = 200 m, *b*_1_ = 2.5°, *b*_2_ = 3° and *c*_1_ = *c*_2_ = 5°. The resulting CDF curves of the location error are as shown in Figure 11. The divided type with six input data subsets provides better location estimation than the composite type. TSA and HLOP lead to less accurate results under this condition, and the proposed algorithm can still give better MS location estimate.

**Figure 12.**
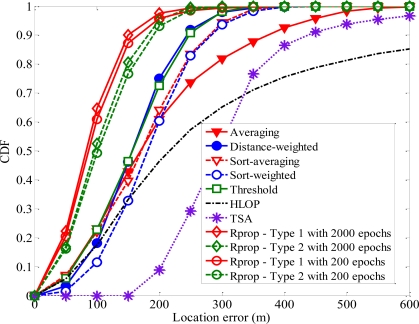
CDFs of the location error of the other different methods and the proposed algorithm with 2,000 and 200 epochs.

Figure 12 shows CDFs of the MS location error for all methods based on a biased uniform random variable model. The proposed algorithm with 2,000 epochs is slightly better than that with 200 epochs. The simulation results show that the positioning precision of the proposed algorithm with only 200 epochs still yield superior performance when compared with TSA, HLOP and the other geometrical positioning methods. Our proposed algorithm with 200 epochs still enhances the performance of MS location estimation effectively.

**Figure 13.**
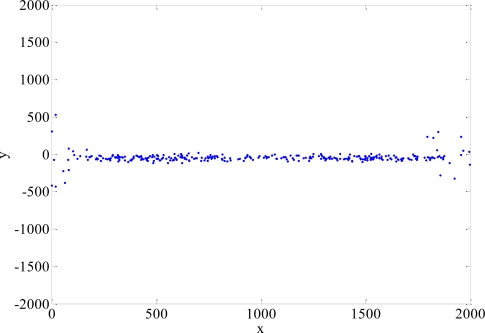
Distribution of the divergence points for TSA.

**Figure 14.**
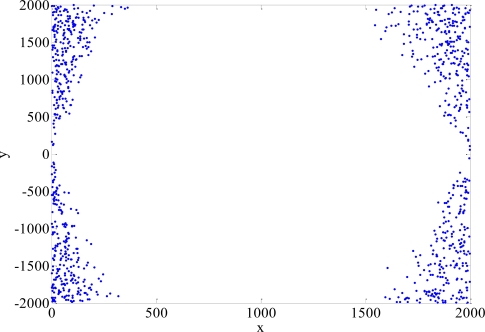
Distribution of the divergence points for HLOP.

When the MS is close to the condition of being aligned with the two BSs, TSA may not converge. HLOP can result in large location errors when the measured angle approaches 90° or 270°. We define the divergence point when the RMS error is above 3,000 m. The distributions of the divergence points of TSA and HLOP are shown in Figures 13 and 14, respectively. The divergence probabilities for different NLOS errors are between 0.32% and 4.66% [17]. The divergence points of TSA and HLOP are not used to calculate the RMS errors and CDF’s in our simulations. Note that the proposed algorithms do not have such divergence problem for this situation. TSA and HLOP won’t be any divergent problems in the case of more than two BSs available to use. No matter what NLOS propagation model is considered, the simulation results show that the proposed algorithms for Rprop can give very accurate results in the MS location estimation after the training period.

## 7. Conclusions

This paper proposes novel Rprop-based algorithm to obtain approximate MS location. We combine both TOA and AOA measurements to estimate the MS location under the condition that the MS is heard by only two BSs. The key issue is to apply Rprop to model the relationship of the remaining feasible intersections and MS location. After training, the proposed algorithm can reduce the effects of NLOS errors and improve MS location performance. One the other hand, the traditional methods of TSA and HLOP may not converge when the MS/BSs have an undesirable geometric layout. The positioning accuracy of the proposed algorithms is hardly affected by the relative position between the MS and BSs. Simulation results show that the convergence performance of the proposed algorithms are quite well and provides the capabilities to explicitly reduce the effects of NLOS errors. In summary, the proposed algorithm can always yield better performance than TSA, HLOP and the geometrical positioning methods for different levels of NLOS errors.
